# Elevated Lead in Drinking Water in Washington, DC, 2003–2004: The Public Health Response

**DOI:** 10.1289/ehp.8722

**Published:** 2007-01-17

**Authors:** Tee L. Guidotti, Thomas Calhoun, John O. Davies-Cole, Maurice E. Knuckles, Lynette Stokes, Chevelle Glymph, Garret Lum, Marina S. Moses, David F. Goldsmith, Lisa Ragain

**Affiliations:** 1 Center for Risk Science and Public Health, Department of Environmental and Occupational Health, School of Public Health and Health Services, George Washington University Medical Center, Washington, DC, USA; 2 Department of Health, District of Columbia, Washington, DC, USA

**Keywords:** biomonitoring, blood lead level, children’s environmental health, drinking water, lead exposure, population surveillance, screening program

## Abstract

**Background:**

In 2003, residents of the District of Columbia (DC) experienced an abrupt rise in lead levels in drinking water, which followed a change in water-disinfection treatment in 2001 and which was attributed to consequent changes in water chemistry and corrosivity.

**Objectives:**

To evaluate the public health implications of the exceedance, the DC Department of Health expanded the scope of its monitoring programs for blood lead levels in children.

**Methods:**

From 3 February 2004 to 31 July 2004, 6,834 DC residents were screened to determine their blood lead levels.

**Results:**

Children from 6 months to 6 years of age constituted 2,342 of those tested; 65 had blood lead levels > 10 μg/dL (the “level of concern” defined by the Centers for Disease Control and Prevention), the highest with a level of 68 μg/dL. Investigation of their homes identified environmental sources of lead exposure other than tap water as the source, when the source was identified. Most of the children with elevated blood lead levels (*n* = 46; 70.8%) lived in homes without lead drinking-water service lines, which is the principal source of lead in drinking water in older cities. Although residents of houses with lead service lines had higher blood lead levels on average than those in houses that did not, this relationship is confounded. Older houses that retain lead service lines usually have not been rehabilitated and are more likely to be associated with other sources of exposure, particularly lead paint. None of 96 pregnant women tested showed blood lead levels > 10 μg/dL, but two nursing mothers had blood lead levels > 10 μg/dL. Among two data sets of 107 and 71 children for whom paired blood and water lead levels could be obtained, there was no correlation (*r*^2^ = –0.03142 for the 107).

**Conclusions:**

The expanded screening program developed in response to increased lead levels in water uncovered the true dimensions of a continuing problem with sources of lead in homes, specifically lead paint. This study cannot be used to correlate lead in drinking water with blood lead levels directly because it is based on an ecologic rather than individualized exposure assessment; the protocol for measuring lead was based on regulatory requirements rather than estimating individual intake; numerous interventions were introduced to mitigate the effect; exposure from drinking water is confounded with other sources of lead in older houses; and the period of potential exposure was limited and variable.

In this article we report the findings of a lead-screening program instituted for residents of the District of Columbia in response to increased lead levels in drinking water in 2003 and 2004. The results are of interest as a population survey of residents, an evaluation of the public health implications of a lead exceedance, and a case study in emergency response to a drinking-water event.

A number of advisories and interventions were introduced at the time in order to reduce exposure and to mitigate any public health risk that would result. Among the responses mounted by the District of Columbia Water and Sewer Authority (DCWASA) and the DC Department of Health (DOH) was a screening program for elevated blood lead levels that targeted young children, pregnant women, and nursing mothers.

Washington, DC, has had a well-documented problem with lead exposure associated with residual lead paint and contaminated house dust in older housing, mostly built before 1950 and never rehabilitated. Lead levels in the blood of children in the district have been falling for many years and continued to fall through the period of elevated lead in the drinking-water distribution system (Stokes et al. 2004).

Recently, in part in response to the situation in Washington and a similar situation in Greenville, North Carolina, Miranda et al. (2006) reported that, on a population basis, blood lead levels in children correlated with the use of chloramine as a disinfection agent, an effect concentrated in older housing stock.

## Case History

In 2002, lead concentrations in treated water supplied by the DCWASA began to rise. Because the increase was small and did not exceed the U.S. Environmental Protection Agency’s (EPA) lead action level (LAL), the significance of this finding as a harbinger of further increases was not appreciated at the time. The increase followed the substitution in water-disinfection treatment from chlorine to chloramines on 1 November 2002, in anticipation of the new Disinfection Byproducts Rule, later published on 4 January 2006 (U.S. EPA 2006a). Thereafter, the rise was obvious and sustained and exceeded the action level, which stipulates that the 90th percentile of samples cannot exceed 15 ppb (equal to micrograms per liter) (Nakamura 2004). At its peak in early 2004, the 90th percentile of homes sampled was 59 ppb; 68% of 6,170 addresses where water was sampled exceeded the LAL of 15 ppb on first-draw samples; and lead concentrations on first draw averaged 14 ppb. During this period, some homes exceeded 300 ppb. Such a large-scale disturbance in lead levels was unprecedented in recent water management history (U.S. EPA 2006b). Subsequently, other cities have reported problems with rising lead levels under similar circumstances (Miranda et al. 2006; U.S. Water News 2004).

The DCWASA serves approximately 2 million wastewater customers and supplies about 500,000 customers in the metropolitan Washington area with 135 million gallons (approximately 520 million liters) of drinking water per day at 130,000 locations. In a unique arrangement reflecting national security concerns dating to the Civil War, the DCWASA purchases finished water from the Washington Aqueduct, which is a division of the Army Corps of Engineers. The Washington Aqueduct draws raw water from the Potomac River, treats it, and sells the finished water wholesale to Arlington County, Virginia, and to the City of Falls Church, Virginia, as well as to the DCWASA, which distributes it throughout the District of Columbia. The District of Columbia is also unique because the Government of the District of Columbia does not have direct regulatory oversight (“primacy”) over the DCWASA for environmental standards. The DCWASA reports directly to U.S. EPA, Region III.

Drinking water supplied to the distribution system is essentially free of lead up to and through the main lines, which typically run down the middle of city streets under the pavement. Smaller service lines conduct the water from the main line to a house, where the service line connects with the interior plumbing, which is usually mostly copper. Lead was standard from the nineteenth century until the 1940s as the material of choice for service lines and continued to be used occasionally until the 1970s, when it was completely replaced by copper, polyvinyl chloride, or other materials. Because lead service lines were once used in all houses, regardless of location, design, or price, they are still present in a wide range of older housing types in District of Columbia, from expensive and desirable homes in affluent neighborhoods to neglected housing in marginal areas.

Lead is subject to leaching under certain conditions of corrosivity. Switzer et al. (2006) assumed, and later confirmed, that the change in disinfection from chlorine to chloramines had altered the leaching of lead from the interior surface of lead service lines, causing lead levels in tap water to rise. (The actual change in water chemistry was later found to be more complicated, as described below.) Other potential sources of lead within the household include solder in joints between copper pipes, older faucets, and certain types of water meters, some of which were previously marketed as “lead free” (Schock 1999). These sources would be subject to the same leaching effect. In the District of Columbia, the lead service line is the responsibility of the utility company from the main line to the property line (the “public” segment of the line) and of the homeowner from the property line to the tap (the “private” segment), including the various other fixtures that may contain lead.

Over the years, most of these lead service lines have been replaced, particularly when houses have been renovated. As a consequence, certain homes have elevated lead levels at the tap and others, which may be on the same street or even next door, do not. An analysis of 7,158 houses that were directly inspected through test pits by the DCWASA, reported to the U.S. EPA in 2005 (in a letter dated 1 August 2005 from the Chief Engineer of DCWASA to Karen Johnson, Chief of the Safe Drinking Water Act Branch), revealed that homes with lead service lines in the District of Columbia are most likely to be on streets with at least one other lead service line (70%), are most likely to be associated with homes built between 1900 and 1933 (81%) or before 1899 (60%, less than those built later because of subsequent renovations), are less likely to have been built between 1934 and 1949 (27%), and are least likely to have been built since 1950 (11%).

Drinking water is regulated under several rules promulgated under the Safe Drinking Water Act (1974), among which the Lead and Copper Rule (LCR) specifically covers lead levels in drinking water (U.S. EPA 1991). This complicated rule has several parts governing sampling (e.g., mandating “first draw” sampling for residences), monitoring strategy, and the prescribed response, which includes public education, notification of responsible officials, measures to reduce personal and family exposure, and initiation of a program to replace lead service lines on public property.

Following the LCR (U.S. EPA 1991), guidance from the U.S. EPA, consultation with the DC Department of Health, and its own contingency plans, in 2003 the DCWASA implemented plans for families living in homes with lead lines or testing above the LAL:

- Advisories were disseminated recommending that water lines should be flushed for 10 min before consuming drinking water.
- Specific advice for limiting exposure to children < 6 years of age and pregnant and nursing women was sent to all households with suspected lead service lines, in the form of flyers prepared in English, Spanish, Korean, Chinese, Vietnamese, and Amharic.
- Filters were distributed to homes with suspected lead service lines and later to all homes with a test result > 15 ppb (the LAL). Replacement filter cartridges were then sent to the same homes at 6-month intervals for the duration of the period of the exceedance, ending in June 2006.
- The board of directors of the DCWASA decided to adopt a voluntarily accelerated program to replace the public segment of all lead service lines in the District of Columbia, exceeding requirements of the LCR (U.S. EPA 1991).
- Homeowners were offered replacement of the private segment of lead service lines on their property, at cost, at the same time that the public segments of the lead service lines were replaced. When the public line is replaced but the private line is not, lead levels are reduced proportionally to the length of pipe replaced but not eliminated.
- Low-cost financing was arranged with a local bank for qualifying property owners who wished to replace the private part of the lead service line on their property. The DC government later made grants available to low-income eligible residents for this purpose.
- The DCWASA offered free water testing to any customer in the distribution area who requested it.

Beginning in August 2004, the DCWASA conducted an Optimal Corrosion Control Treatment (OCCT) study, as required by the LCR (U.S. EPA 1991), to evaluate various methods of corrosion control in their distribution system. The evidence from the OCCT study and other investigations suggest that reduction in the residual chlorine level, a result of the switch to chloramines, changed the reduction–oxidation (redox) potential at the interior surface of the pipe, changing the equilibrium point and causing release of previously stable lead from the surface (Switzer et al. 2006). The physicochemical basis for this process will be described in a forthcoming report (Cadmus and U.S. EPA, unpublished data). After consultation with the stakeholder agencies, the DCWASA, the U.S. EPA, the DC DOH, and the Washington Aqueduct, orthophosphate—a commonly used passivating agent that is also used in commercial beverages—was added to the water. This wholly conventional water treatment has had the expected effect of reducing lead levels in the distribution system (Schock 1999).

From the fall of 2003, the DCWASA embarked on a massive program of replacing lead service lines, as required by the LCR (U.S. EPA 1991) and accelerated by the the DCWASA board of directors. There were 1,016 priority replacements for three (overlapping) criteria, 92% in houses where at least one resident was known to be at risk (a child < 6 years of age or a pregnant or nursing woman), 7.2% because a child was enrolled in day care (determined as part of the lead-screening outreach, described below), and 5.1% because a child living in the house had been determined to have an elevated blood lead level. In only 22.8% of priority replacements did the owners of the houses have the private segment of the lead service line replaced at the same time as the public segment.

The lead elevation has now abated. The DCWASA is now in compliance with the LCR (U.S. EPA 1991), and health advisories associated with the elevation were lifted in January 2006 by the DC DOH.

## Materials and Methods

Blood lead determinations are readily accessible to residents of the District of Columbia through a network of public and private facilities. Blood lead testing is mandatory for DC children at 1 and 2 years of age. Elevated blood lead levels are also reportable in the District of Columbia to the DC DOH Childhood Lead Poisoning Prevention Program. Because of concern over the issue of lead in drinking water, greater capacity was required to handle the demand for screening and also to provide counseling, educational, and referral services as needed. The DC DOH therefore developed a program to supplement the existing clinical screening program at no cost to DC residents. The program involved expanded clinic hours at five existing community outpatient clinics (Anacostia, Congress Heights, the former DC General Hospital site, Greater Southeast Hospital, and 51 N Street, NE) and blood drawing on site at schools, licensed child-care centers (which have an allowable maximum of five children in care), and the Children’s National Medical Center. The screening program began on 3 February 2004 and was discontinued on 2 August 2004. This special program was funded by the DCWASA at a total cost of $1 million. Passive monitoring through mandatory pediatric lead screening and voluntary testing for all DC residents continues at the five originally designated centers, as before.

The program was extensively publicized in local media and health services and by direct outreach to parents whose children were enrolled in day care. A letter recommending screening and outlining the public health advisories was sent individually on 26 February 2004 from the DC DOH to residents of 23,000 homes identified as having a high probability of being served by lead service lines. Notices were also sent home to parents of students in DC public schools.

Lead screening was also offered on-site at 84 of 155 active child-care facilities with known or suspected lead service lines in the District of Columbia (out of a total of 233): 36 child-care facilities had made arrangements for screening to be conducted by a private physician, 28 had no children enrolled at the time, 6 were not tested because parents refused, and 1 facility refused. On 11 May 2004, following monitoring results that showed increased lead levels in water at some school taps, the screening program was extended to pupils in the DC city schools, eventually reaching 32 public (as opposed to charter) schools (Washington has 167 elementary schools) and 3 charter schools (out of 34).

For purposes of analysis, we identified two groups: *a*) the “target population,” which we defined as children 6 months to 6 years of age (referred to as “children < 6 years of age”) and women who were pregnant or nursing; and *b*) the population “outside the target population,” which we defined as all others for whom testing was requested.

Blood lead levels were measured in the public health laboratory of the DC DOH by graphite furnace atomic absorption spectrometry. Elevated blood lead levels were defined as those > 10 μg/dL, the level of concern adopted by the Centers for Disease Control and Prevention (CDC 1991).

We analyzed results of the determinations for all confirmed and probable residents of Washington, DC, for the target population, and for various subgroups. A few subjects who gave DC addresses but actually lived in Maryland (which is served by a different drinking-water distribution system and was not affected by chloramination) were screened inappropriately; they have been omitted from the analysis.

The homes of all children and adults with elevated blood lead levels were investigated by the DC DOH. The results of public health investigations in the home for the elevated levels for adults and children were reviewed. To protect confidentiality, results were not communicated to the public in sufficient detail to identify house addresses, institutions, or neighborhoods.

The study was undertaken as a public health intervention by the DC DOH rather than a research project and was therefore not subject to internal review board review. The DC DOH complied with all applicable requirements of U.S. and international standards, and participants (or legal guardians in the case of minors) gave written informed consent before having blood drawn.

A subset of 177 houses with water lead levels of > 300 ppb was identified by the DCWASA through its sampling program, and the residents were invited to participate in the lead-screening program.

The records of children screened during the special lead-screening program were entered into the general database for pediatric blood lead levels in the DC DOH. Data on housing has been transferred to a newly created DC Department of Environment. The DC DOH identified 2,482 children < 6 years of age when tested in 2004, of which 107 cases had paired values of blood lead and first-draw water lead concentration. A correlation analysis was performed on this data set.

We obtained a data set from the DCWASA that was prepared at the request of the U.S. EPA in 2005; this data set included 71 individual children (at 67 addresses) with blood lead levels of ≥ 10 μg/dL for which paired blood lead levels (in the case of multiple draws, the highest) and water concentration were available. An address identifier was available for each individual. It is not possible to know how many subjects appear in both data sets because individual cases can only be identified by a match on exact numerical value of both parameters. However, in a preliminary check of the seven highest blood lead levels (those ≥ 10 μg/dL on the DC DOH data set), only three cases appeared to be represented on both lists. We performed a correlation analysis on this data set on all data points, including repeated measures on each individual, by address, and on the highest reported value among multiple values of either blood lead or water lead concentration, whether first draw or second draw (10 min running tap) or multiple samples.

## Results

**Figure 1:**
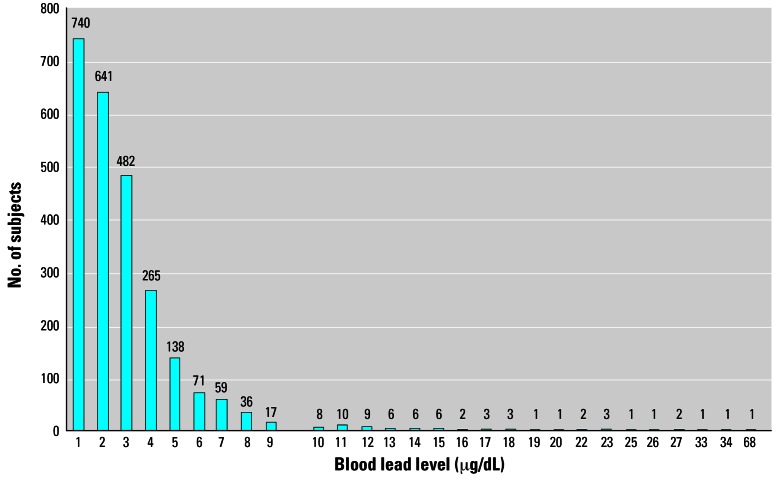
Distribution of blood lead levels for the target group (children < 6 years of age, pregnant women, and nursing women) tested during the 8-month period in 2004 (as of 12 July 2004).

Table 1 provides a profile of participants in the lead-screening program. During the period 3 February 2004 to 12 July 2004, a total of 6,834 persons were screened for blood lead level. The mean age of those tested was 21.01 years. The youngest was < 1 year and the oldest was 99 years of age, with a mean age of 21 and a median of 9 years (no resident who requested testing was turned away during this period). Of these subjects, 2,516 were within the target population: 2,342 (93.1%) children < 6 years of age, 96 (3.8%) pregnant women, and 78 (3.1%) women who were nursing. Figure 1 presents the distribution of blood lead levels for the target population.

Of the 2,342 children < 6 years of age, 361 (15.4%) had lead service lines supplying water to their residences. Of these, 19 (5.3%) had elevated blood lead levels. Among the subset of 1,098 pupils screened in the DC city schools, 232 were children < 6 years of age; of these, 15 (6.5%) had lead service lines supplying their residences, and 1 of the 15 had an elevated blood lead level. Another subset consisted of 155 children screened in child-care centers, 2 of whom had elevated blood lead levels.

The characteristics of all cases with elevated blood lead levels are presented in Table 2. Children from 6 months to 6 years of age constituted 2,342 of those tested. Of these children, 65 (2.8%) had blood lead levels > 10 μg/dL, and all but 1 had a level < 45 μg/dL, a level that may be associated with clinically symptomatic lead poisoning, That 1 child had a level of 68 μg/dL and was hospitalized. A decision to treat by chelation was deferred because a repeat blood lead determination showed that the level was falling. A source of lead exposure unrelated to either lead paint or water has been identified in that case but has not been revealed in order to protect the confidentiality of the family. No other child or adult with elevated blood lead levels required medical treatment. Most of the children with elevated blood lead levels (*n* = 46; 70.8%) did not live in homes with lead service lines.

**Figure 2:**
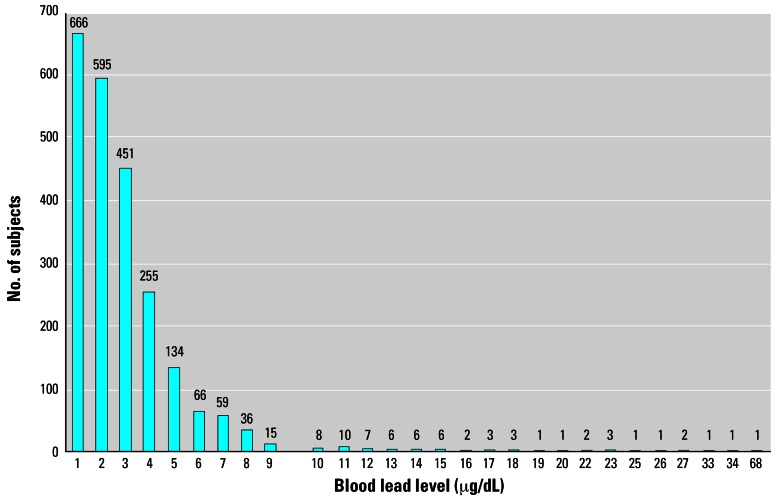
Distribution of blood lead levels among children < 6 years of age who were residents of the District of Columbia and were tested in the screening program during the 8-month period in 2004 (as of 12 July 2004).

Figure 2 presents the blood lead levels for children < 6 years of age. Twelve children 6–15 years of age had elevated blood lead levels (ranging from 10 to 19 μg/dL). Not all of the members of the target group who had elevated blood lead levels were children. Two nursing mothers had blood lead levels > 10 μg/dL. None of the 96 pregnant women tested showed elevated blood lead levels.

In every case in which the blood lead level exceeded 10 μg/dL in a subject in the target population, an investigation of the homes was conducted. Most identified at least one source of lead exposure other than drinking water, usually peeling lead paint and dust. Two cases remain in dispute because a source has not been positively identified, but there is no evidence that either is water related. This investigation is continuing.

Among those children < 6 years of age who had blood lead levels < 10 μg/dL, the blood lead level (mean ± SD) for the 344 children who lived in homes with lead service lines was 3.28 ± 2.05 μg/dL compared with 2.60 ± 1.69 for children living in homes without lead service lines, a statistically significant difference (*p* < 0.05).

Among the 4,318 residents screened who were outside the target population, 4 had blood lead levels > 25 μg/dL, the level of concern for adults (CDC 1991). None were symptomatic. Two had lead service lines and two did not.

Table 3 presents a summary of blood lead levels of all participants and for children < 6 years of age. The geometric mean blood lead level for children < 6 years was 2.3 μg/dL, compared with a national geometric mean value for children 1–5 years of age in the United States of 1.9 μg/dL overall, 2.8 μg/dL for non-Hispanic African Americans, and 3.7 μg/dL for children of families with low income, as derived from data collected during the National Health and Nutrition Examination Survey in 1999–2002 (Schwemberger et al. 2005; Stokes et al. 2004).

Table 4 presents a breakdown of all children < 6 years of age whose blood lead levels fell below the CDC level of concern and summary measures showing that children who lived in homes with lead service lines had a higher blood lead level (*p* < 0.05). The geometric mean blood lead of children who lived in houses with lead service lines was 3.28 μg/dL [95% confidence interval (CI), 3.06–3.50] compared with 2.60 μg/dL (95% CI, 2.53–2.67) for those living in houses without lead service lines.

Of the 177 homes with > 300 ppb lead in drinking water, the residents or owners of 44 could not be contacted after multiple home visits and telephone calls; the residents of 14 had their lead levels tested privately; the residents of 10 homes refused to participate; and 210 residents of 119 houses participated in the screening program. None had a blood lead level > 10 μg/dL.

In the data set obtained from the DC DOH on 2,482 blood lead determinations, we found 107 children with paired blood lead level and first-draw water lead concentrations. Of these, 7, and an additional 52 for which paired values were not available, were at ≥ 10 μg/dL. The range of blood lead levels was 1–68 μg/dL in the total list and 1–25 μg/dL among the 107 with paired values. The range of first-draw water lead concentration was 1.04–310 ppb. There was no correlation (*r*^2^ = –0.03142).

The data set obtained from the DCWASA included 71 mostly additional individuals for which the highest blood lead level and highest water lead concentration included 20 individuals with blood lead levels of 10 μg/dL and ranging up to 53.6. The range of highest water lead concentrations was 0–584. There was no correlation: *r*^2^ = –0.0856 for individuals, *r*^2^ = –0.05639 for all data points, and *r*^2^ = –0.09728 for all addresses.

## Discussion

The present study is not an ecologic study of blood lead levels in association with lead concentrations in drinking water. Rather, it is a case study of a prolonged incident of elevated lead in which massive public health interventions may have mitigated the potential effects of exposure.

Exposure measurement is not easily comparable among studies of lead in drinking water. Under the LCR (U.S. EPA 1991), lead in tap water is measured on “first draw,” representing water that has sat overnight in pipes serving the house before any flushing has occurred. The measurement therefore represents the presumed maximum, reflecting leaching from lead-containing pipes and joints overnight, that might occur with first use of tap water in the morning, not the steady-state concentration during continuous flow, which is measured by other protocols. The first-draw sampling method has resulted in estimates of lead exposure 39% higher than the convenience sampling method of measuring lead in “kettle” water that is actually consumed in a household (Lacey et al. 1985).

The effect of lead in drinking water may have been mitigated due to the effectiveness of the interventions undertaken, the transient nature of first-draw exposure, the diversity of sources of fluid intake, or because lead in drinking water is not a major source of intake except for formula-fed infants (Gulson 1997; Hilbig et al. 2002; Lacey et al. 1985). As a consequence, the present study should not be considered as evidence that lead in drinking water is a negligible source of lead intake or that it is never associated with elevated blood lead levels.

Young children are at greatest risk for health outcomes associated with lead exposure because their nervous systems are developing rapidly (Slikker 1994; Stokes et al. 1998). The level of concern adopted in 1991 by the CDC as a guideline for prevention is 10 μg/dL (CDC 1991). Although much lower than levels known to be associated with clinically symptomatic lead poisoning, the CDC level of concern is not completely protective against neurobehavioral outcomes (Bellinger and Needleman 2003; Canfield et al. 2003). In population studies, blood lead levels < 10 μg/dL have been associated with reduced mental capacity (as measured by intelligence testing), reduced academic performance, and increased risk of aggressive behavior and mental illness—effects that have demonstrated exposure–response relationships with no threshold (Lanphear et al. 2000; Nevin 2000; Rogan and Ware 2003). Pediatricians and public health authorities therefore advise that children’s exposure to lead from all sources be reduced as much as feasible to keep blood lead levels as low as possible during childhood (Campbell and Osterhoudt 2000; Landrigan 2000; Todd et al. 1996).

Drinking water has been estimated to contribute about 7% of the intake of lead in U.S. children, overall [Agency for Toxic Substances and Disease Registry (ATSDR) 1999]. In toxicokinetic models, the U.S. EPA assumes that 50% of lead in drinking water is absorbed by children (U.S. EPA 2002) and that, as a default assumption, drinking water contributes 20% of an individual’s lead intake, while stipulating that “these default values may not be appropriate for specific applications” (U.S. EPA 1994). These estimates of absorption and general intake are averages and do not apply to children exposed to a known source of lead, and they do not apply to infants who consume formula made from tap water (Gulson 1997; Hilbig et al. 2002).

There are few studies that correlate blood lead levels with lead in drinking water, and those of greatest interest use exposure metrics other than the first-draw sampling protocol required under the LCR (Lacey et al. 1985). Studies associating highly elevated lead in drinking water on the order of 1,000 ppb (measured as a convenience sample from household kettles) with elevated blood lead levels in individual women residents of Ayr, Scotland, suggested that marked reductions occurred following measures to mitigate exposure (from 21 to 13 μg/dL) (Sherlock et al. 1984). Studies of decline in median blood lead in young women > 20 years of age in Glasgow, Scotland, also showed a decline over decades, but exposure from other sources may also have been reduced (Watt et al. 2000). More recently, investigators in Hamburg, Germany, demonstrated a close correlation between lead in tap water (measured as an average of first-draw, consumption, and flushed samples) and blood lead levels in non-smoking women, and marked reductions following interventions designed to avoid exposure in tap water (Fertmann et al. 2004). In each case, the metric for lead in tap water was not the same as in the present study.

In the present study, lead concentrations were measured on first-draw samples, as required by the LCR (U.S. EPA 1991). Residents of houses with elevated lead in tap water may consume this water, for example, first thing in the morning, but relatively brief flushing (much less than 10 min) dramatically reduces the lead concentration. Adults, especially, also diversify their fluid intake and do not drink tap water exclusively (Heller et al. 2000). Except for nursing infants whose formula is prepared primarily from first draw tap water, the average concentration in water consumed through the day is therefore much less than the first-draw concentration, when the first-draw sample is elevated. This may be why adults who drink first-draw tap water every morning at levels as high as 3,000 ppb did not have higher blood lead levels than those who did not (Goldberg 1974).

There is no evidence for a strong selection bias against residents at lower risk in the screening program; rather, it is likely that a disproportionate number of residents at higher risk were screened and that their screening was motivated by parental concern. The 2,342 children < 6 years of age who were screened represent 5.1% of an estimated 45,549 children in the District of Columbia in that age group. Many of those not screened who were 1 or 2 years of age at the time would have already had recent mandatory blood lead determinations, and their parents or guardians, knowing the results, may not have sought a repeat test. The District of Columbia covers a small area (260 km^2^), and the testing centers were readily accessible by public transportation and even by foot. The distribution of subjects mapped as of March 2004 closely matched both the distribution of housing dating before 1950 and density of children < 6 years of age (DC DOH, unpublished data). (The one exception was base housing for Bolling Air Force Base, where there are many children < 6 years of age but construction is recent and military health services are available.)

Recruitment for this study took place through many channels and was reinforced by media coverage of the issue, which was close to saturation at the time, as tracked by the DCWASA Office of Public Affairs using the following indicators. There were 46 stories on the subject in the first 10 months of 2004 on one popular radio station (WJLA) alone, 31 stories in the *Washington Post*, 21 stories in one local newspaper (*The Northwest Current*), and 9 stories in the *Washington Afro-American*. Direct information was also provided during this period to thousands of callers to the DCWASA customer service line, which served 51,031 calls during the 10 weeks of the first extensive news coverage of the issue in late January 2004: 5,429 calls the first week and similar numbers thereafter until tapering in weeks 8 and 9, peaking at 8,556 in week 5, compared with a normal volume of ≤ 40 calls/week. The story was sufficiently prominent that it is unlikely that any families in the District of Columbia with young children were completely unaware of the issue or the availability of the screening program.

Although the percentage of all children < 6 years of age with blood lead levels above the level of concern who were living in homes with lead service lines (5.2%) is larger than the percentage of all children in this age group who were living in homes without lead service lines (2.3%), it is misleading to make a direct comparison. The presence of lead service lines to a house is a marker for age of the house and unrehabilitated status, and therefore indicates higher risk for exposure to lead in paint, soil, and fixtures unrelated to drinking water (Stokes et al. 2004). Homes without lead service lines may be either new homes, which are unlikely to have had interior lead paint when built, or old homes in which the service line may have been replaced during a general refurbishing that included lead abatement.

The District of Columbia Plumbing Code (2003) has required since at least the 1980s that lead service lines be replaced in houses undergoing substantial rehabilitation, defined as involving cumulative repairs or alterations to ≥ 50% of the water system. The requirement was enforced by the permitting process, which was enforced by a bond on master plumbers. A 1990 consultant firm’s report (Weston 1990) estimated that more than half of building renovations done at that time included replacement of the lead service line and that lead had not been used in replacement lines since the mid-1940s. Until 1992, these replacements were usually performed by the Water and Sewer Utility Administration (WSUA), the city agency that preceded the DCWASA. However, after a staffing cut at WSUA in 1992, replacements were often done privately at the owner’s expense, especially when the homeowner was in a hurry to sell the house (DCWASA, personal communication). As a consequence, houses in the District of Columbia that still have lead service lines are those that have not been extensively rehabilitated since the 1940s and are therefore unlikely to have been remediated for other lead sources, including lead paint, lead-containing solder, and lead-containing brass in water meters.

In collaboration with the CDC, the DC DOH reported blood lead levels of children who lived in homes with and without lead service lines in 2004 and compared them with historical trends (Stokes et al. 2004). Children living in homes with lead service lines showed a decline in frequency of elevated blood lead over time at the same rate as children in homes without lead service lines. The downward trend was steady and uniform except for a slight increase between 2000 and 2001, after which levels resumed their decline through 2003. The discontinuity between 2000 and 2001 came too early, and the distribution was too restricted geographically, to suggest an effect of drinking water. Furthermore, in the cross-sectional study conducted in March 2004, none of the residents of homes with > 300 ppb lead in drinking water showed an elevated blood lead level.

The present study cannot be reconciled with the findings of Miranda et al. (2006) from Wayne County, North Carolina, because of differences in study design and case history. Miranda et al. (2006) found a correlation at the population level between chloramination of drinking water and blood lead levels of children under 6 years of age. The ecologic study design used by Miranda et al. (2006) examined correlations at a population level and found an association with chloramination, a stronger association with year of construction of the house at the address where the child was living, an even stronger relationship with household median income, and a strong interactive term between year of construction and use of chloramines. They attribute this association to the possible action of chloramines on lead pipes in older homes, but the design of their study cannot rule out confounding with housing quality. However, no sampling data, ranges, or representative values of lead in water are provided and no ranges or distributions are provided for blood lead levels. In the absence of individual data, implications for risk on a personal level cannot be assessed. Their findings may apply to a different range or distribution of values than those observed in the present study. In contrast, their data may reflect the experience of an unprotected population, without the mitigating effect of the public health measures introduced in Washington, DC, or a population with fewer significant sources of lead exposure than in Washington.

There appears to have been no identifiable public health impact from the elevation of lead in drinking water in Washington, DC, in 2003 and 2004. This may reflect effective measures to protect the residents, as 153 reported compliance with recommendations to filter their drinking water. However, the screening program developed in response to the issue uncovered the true dimensions of a continuing problem with sources of lead in homes (Breysse et al. 2004). The incident did uncover an ongoing and serious problem of lead exposure from other environmental sources in the community, which were associated with elevations in blood lead in individual children.

The present study cannot be used to correlate lead in drinking water with blood lead levels directly because *a*) it is based on an ecologic rather than individualized exposure assessment; *b*) the protocol for measuring lead was based on regulatory requirements rather than estimating individual intake; *c*) numerous interventions were introduced to mitigate the effect; *d*) exposure from drinking water is confounded with other sources of lead in older houses; *e*) the period of potential exposure was not uniform among houses; and *f* ) actual exposure was variable for individual residents. It is of value as a population survey of residents, an evaluation of the public health implications of a lead exceedance in the face of an appropriate response, and as a case study in emergency management of a drinking-water event.

The public health objective remains to reduce lead intake further from all sources, to lower mean blood levels in DC (and all) children still further, and to eliminate individual cases of elevated blood lead levels, as determined by the current, or any future revised, CDC level of concern (Campbell and Osterhoudt 2000).

## Figures and Tables

**Table 1:** Performance and results of the screening program.

Characteristic	No. (%)
Total population screened	6,834 (100)
Total no. screened within target population	2,516 (36.8)
Children < 6 years of age	2,342 (93.1)
Children < 6 years of age in homes with lead service lines	361 (15.4)
Pregnant women	96 (3.8)
Nursing mothers	78 (3.1)
No. screened outside target population	4,318 (63.2)
Children 6–15 years of age	1,834 (42.5)
Total tested within DC city schools (of total screened)	1,098 (16.1)
Children < 6 years of age	232 (21.1)
Children < 6 years of age in homes with lead service lines	15 (6.5)
Children < 6 years of age living in homes without lead service lines	217 (93.5)
Children 6–15 years of age	812 (74.0)
Adolescents > 15 years of age	37 (3.4)

Excludes participants confirmed not to have been residents of the District of Columbia at the time of service.

**Table 2:** Results of the screening program in terms of elevations from the level of concern.

Characteristic	No. (%)
Total children and adolescents (≤ 17 years of age) with blood lead levels ≥ 10 μg/dL	77 (100.0)
Children < 6 years of age (percent of all children screened)	65 (84.4)
Children < 6 years of age living in homes with lead service lines	19 (29.2)
Children < 6 years of age living in homes without lead service lines	46 (70.8)
Children 6–15 years of age	12 (15.6)
Total adults (≥ 18 years of age) with blood lead levels ≥ 25 μg/dL	4 (100.0)
Total pregnant women with blood lead levels ≥ 10 μg/dL	0 (—)
Total nursing mothers with blood lead levels ≥ 10 μg/dL	2 (100.0)

**Table 3:** Results of the screening program in terms of blood lead levels.

Characteristic	Value
Age distribution of all participants (years)	
Range	< 1–99
Mean	21
Median	9
Blood lead levels of all 6,834 participants (μg/dL)	
Range	1–68
Geometric mean	2.3
Median	2
Mode	1
Blood lead levels of children < 6 years of age (μg/dL)	
Range	< 1–68
Geometric mean	2.3
Median	2
Mode	1

**Table 4:** Results of the screening program for children < 6 years of age with blood lead levels below the CDC level of concern (10 μg/dL).

Blood lead level (μg/dL)	Living in houses with lead service lines	Living in houses without lead service lines
1	75	621
2	67	557
3	75	396
4	50	212
5	28	110
6	15	52
7	14	45
8	15	21
9	3	12
10	2	6
Total no. (%)	344 (14.5)	2,032 (85.5)
Mean ± SD (95% CI)	3.28 ± 2.05 (3.06–3.50)	2.60 ± 1.69 (2.53–2.67)
